# Effects of polyethylene oxide particles on the photo-physical properties and stability of FA-rich perovskite solar cells

**DOI:** 10.1038/s41598-022-15923-y

**Published:** 2022-07-27

**Authors:** Richard K. Koech, Yusuf A. Olanrewaju, Reisya Ichwani, Moses Kigozi, Deborah O. Oyewole, Omolara V. Oyelade, Dahiru M. Sanni, Sharafadeen A. Adeniji, Erika Colin-Ulloa, Lyubov V. Titova, Julia L. Martin, Ronald L. Grimm, Abdulhakeem Bello, Oluwaseun K. Oyewole, Esidor Ntsoenzok, Winston O. Soboyejo

**Affiliations:** 1 Department of Materials Science and Engineering, African University of Science and Technology, Km. 10 Airport Road, Abuja, Nigeria; 2 Department of Mechanical Engineering, Worcester Polytechnic Institute, 100 Institute Road, Worcester, MA 01609 USA; 3 Department of Mathematics, Physics and Computing, Moi University, P.O Box 3900-30100, Eldoret, Kenya; 4 Program in Materials Science and Engineering, Department of Mechanical Engineering, Worcester Polytechnic Institute, 100 Institute Road, Worcester, MA 01609 USA; 5 Department of Theoretical and Applied Physics, African University of Science and Technology, Km 10 Airport Road, Abuja, Nigeria; 6 Department of Physics, Worcester Polytechnic Institute, 100 Institute Road, Worcester, MA 01609 USA; 7 Department of Chemistry and Biochemistry, Life Science and Engineering Center, Worcester Polytechnic Institute, 100 Institute Road, Worcester, MA 01609 USA; 8 Centre for Cyber Physical Food, Energy and Water System (CCP-FEWS), Electrical and Electronic Engineering Science, University of Johannesburg, Johannesburg, South Africa; 9 CEMHTI-CNRS Site Cyclotron, 3A rue de la férollerie, 45071 Orléans, France; 10 Department of Biomedical Engineering, Gateway Park Life Sciences and Bioengineering Center, Worcester Polytechnic Institute, 60 Prescott Street, Worcester, MA 01609 USA

**Keywords:** Condensed-matter physics, Materials for devices, Materials for energy and catalysis

## Abstract

In this paper, we use Polyethylene Oxide (PEO) particles to control the morphology of Formamidinium (FA)-rich perovskite films and achieve large grains with improved optoelectronic properties. Consequently, a planar perovskite solar cell (PSC) is fabricated with additions of 5 wt% of PEO, and the highest PCE of 18.03% was obtained. This solar cell is also shown to retain up to 80% of its initial PCE after about 140 h of storage under the ambient conditions (average relative humidity of 62.5 ± 3.25%) in an unencapsulated state. Furthermore, the steady-state PCE of the PEO-modified PSC device remained stable for long (over 2500 s) under continuous illumination. This addition of PEO particles is shown to enable the tuning of the optoelectronic properties of perovskite films, improvements in the overall photophysical properties of PSCs, and an increase in resistance to the degradation of PSCs.

## Introduction

Perovskite solar cell (PSC) is a nascent low-cost solar cell technology that has caused a paradigm shift in the field of photovoltaics. This is because of their potential to provide efficient solar harnessing systems with low energy payback times^1–3^. The superior photovoltaic (PV) characteristics of PSCs originate from the excellent optoelectronic properties of their organo-metallic halide perovskite-based photoactive layers^4,5^. Motivated by these properties, multi-faceted research efforts have been expended towards improving their processing conditions, device architecture, and material properties^6,7^. This has led to a rapid evolution in their power conversion efficiencies (PCEs) to the currently recorded values that rival those of the mature crystalline silicon-based PV solar cells^8^.

Unlike the conventional crystalline silicon-based PV solar cells, PSCs are still faced with the problem of degradation when exposed to the conditions in the environment. The ongoing research efforts are directed towards addressing this challenge while trying to increase the PCEs to higher levels^9^. Among the several approaches that have been explored, those that aim at engineering both the active layer (AL) and the interface with the charge transport layers (CTLs) have been cited as the most instrumental routes to achieve improvements in the PCEs, and the long-term stability of PSCs^10–13^. The improvements are attributed to the suppression of defects in the bulk of the perovskite film and the interfaces with the CTLs, which are usually difficult to avoid due to the solution-based processing of perovskite films^14,15^. These defects, not only act as recombination centers, but they also provide degradation pathways in perovskite films thus contributing to the loss of charge carriers and performance degradation^16,17^. Therefore, in order to suppress the power loss and degradation mechanisms in PSCs, in-situ morphological control in the AL is a quintessential step^14,18,19^.

Additive engineering can achieve this goal as well as modulate the interfacial properties of the entire PSCs^20,21^. Polymeric additives incorporated into perovskite precursors have been shown to aid the homogeneous nucleation and crystallization of perovskite films leading to the formation of large perovskite grains with reduced grain boundaries^22,23^. Furthermore, the functional groups present in polymeric compounds enable them to coordinate and cross-link well with the uncoordinated metal ions and other atomic species in perovskite film thus reducing surface defects, increasing the charge carrier lifetime, reducing ion migration, and suppressing moisture ingress in perovskite films^23–25^.

Polymers can also modulate the interfacial barriers at the interfaces between the AL and charge transport layers (CTLs). In particular, ethylene-based polymeric additives such as polyethylene oxide (PEO) not only act as cross-linking and crystallization inhibition agents but they also tune the interface energetics, suppress ion migration, and improve the tolerance of perovskite films to degradation agents^23,26–28^. These properties of PEO have recently been used to modify both the perovskite bulk and the interface properties in PSC leading to improvement in PCE and stability^29–31^. However, there are very few studies focusing on the impact of PEO on the microstructure, electronic band structure as well as charge carrier, and the excited state dynamics of FA-rich mixed halide perovskite films.

In this work, we use a long-chain PEO polymer to passivate defects in the perovskite film, modulate the interfacial energetics and improve the PV performance parameters of a regular planar PSC. Unlike previous studies which incorporated PEO into the perovskite film mainly through the single-step deposition process or as interlayer between the AL and the CTLs, the PEO additive in this work was incorporated via a two-step deposition process. Furthermore, chlorobenzene was used as a solvent to dissolve the PEO and the PEO solution was mixed with the organic components of perovskite precursors before spin coating on the lead iodide layer and annealing them to form perovskite thin films. With this procedure, large-grained perovskite films with improved optoelectronic properties were formed, enabling the fabricated PEO-modified PSC with improved PCE and stability. The champion device containing 5 wt% of PEO showed the highest PCE of 18% with improved resistance to light and moisture-induced degradation in performance. This study provides important information on the role of PEO additives in the fabrication of efficient and stable PSCs.

## Results and discussion

**Figure 1:**
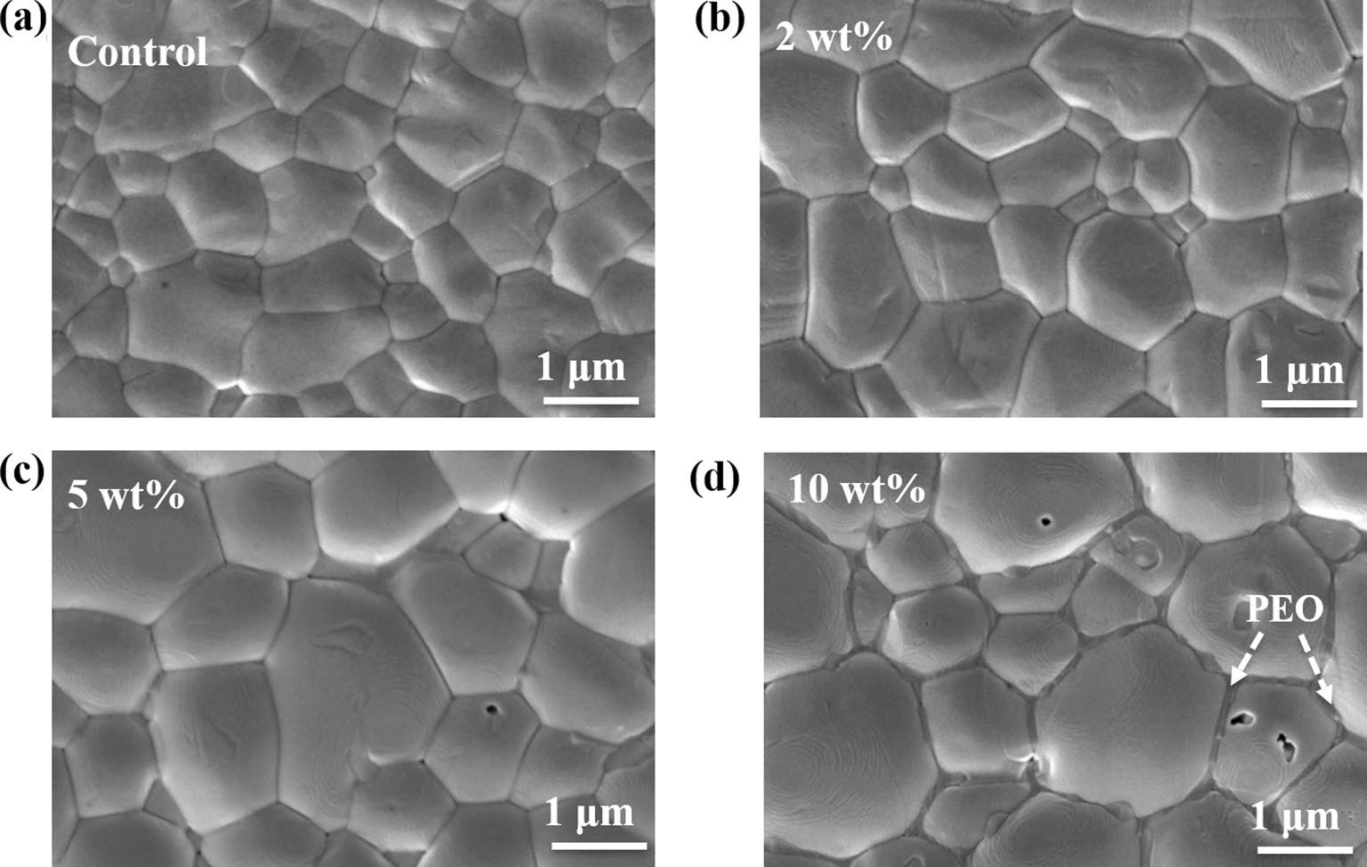
SEM top surface images of perovskite films deposited on FTO substrate with (**a**) 0, (**b**) 2, (**c**) 5 and (**d**) 10 wt% of PEO.

Controlling the morphology of perovskite film is an important route to obtain large and densely packed grains with reduced defect densities that will enable the fabrication of PSCs with improved performance characteristics^32^. The PEO additive incorporated into the perovskite organic precursor solution was able to tune the morphology of the film, as shown in the SEM micrographs in Fig. 1a–d. It is evident that the morphology of the perovskite films improves as the proportion of PEO increases up to 5 wt%. Thereafter, a slight change in the film morphology is observed. At 10 wt% of PEO, the perovskite film appears less compact and the PEO is seen primarily along the grain boundaries.

**Figure 2:**
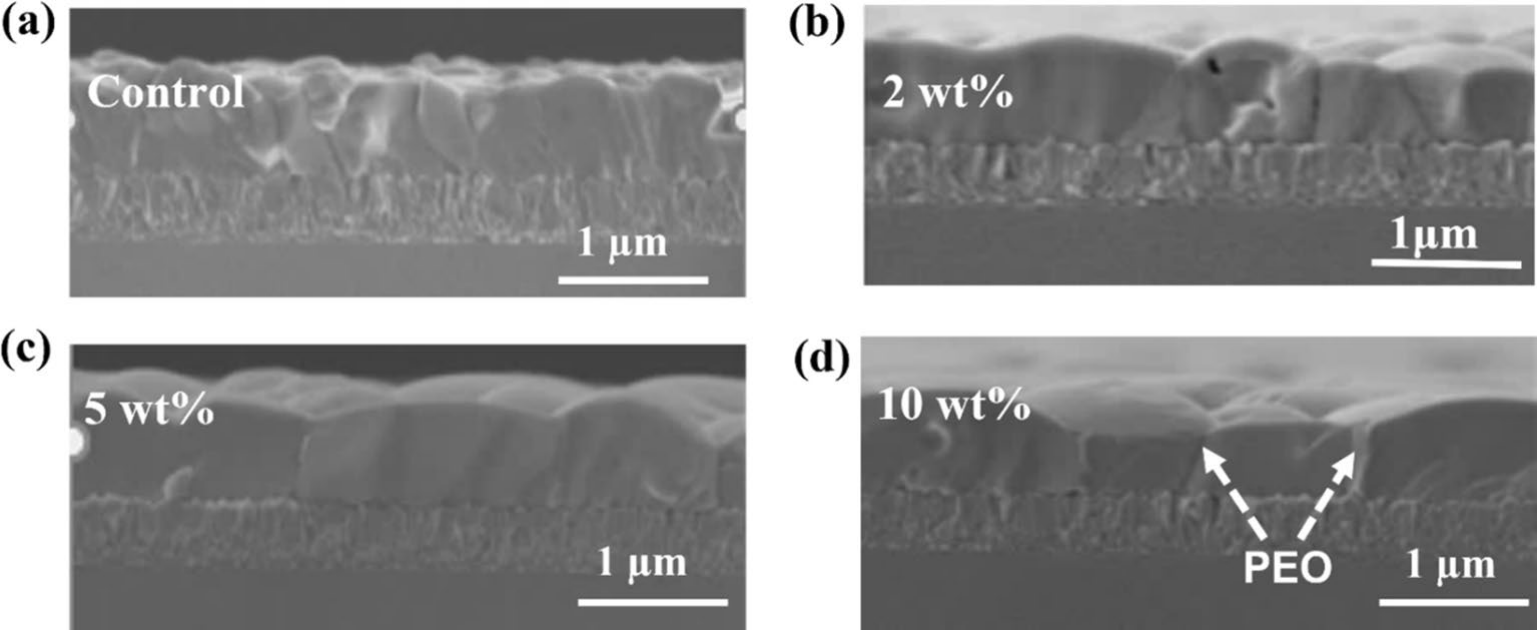
SEM cross-sectional images of perovskite films on FTO substrate with (**a**) 0, (**b**) 2, (**c**) 5 and (**d**) 10 wt% of PEO.

An analysis of the grain sizes of the different films reveals that the average grain size increased with PEO content up to 5 wt% and a slight decrease is observed when the percentage weight of PEO increased to 10. Figure S1 in the supporting information shows the grain size distribution for the perovskite films at different PEO content. It is seen that the grain size increased from an average value of 0.8 ± 0.4 μm for the pure perovskite film to 1.3 ± 0.5 μm for the perovskite film containing 5 wt% of PEO and later decreased to about 1.2 ± 0.5 μm for the film with 10 wt% of PEO. Similarly, the cross-sectional SEM images in Fig. 2a–d show that the grain sizes increased longitudinally with the weight proportion of PEO up to 5 wt%. The available grain boundary areas are therefore greatly reduced when 5 wt% of PEO is introduced into the perovskite film. This reduces the non-radiative recombination and charge carrier trapping centers in the active layer which is important in PCE improvement in PSCs.

**Figure 3:**
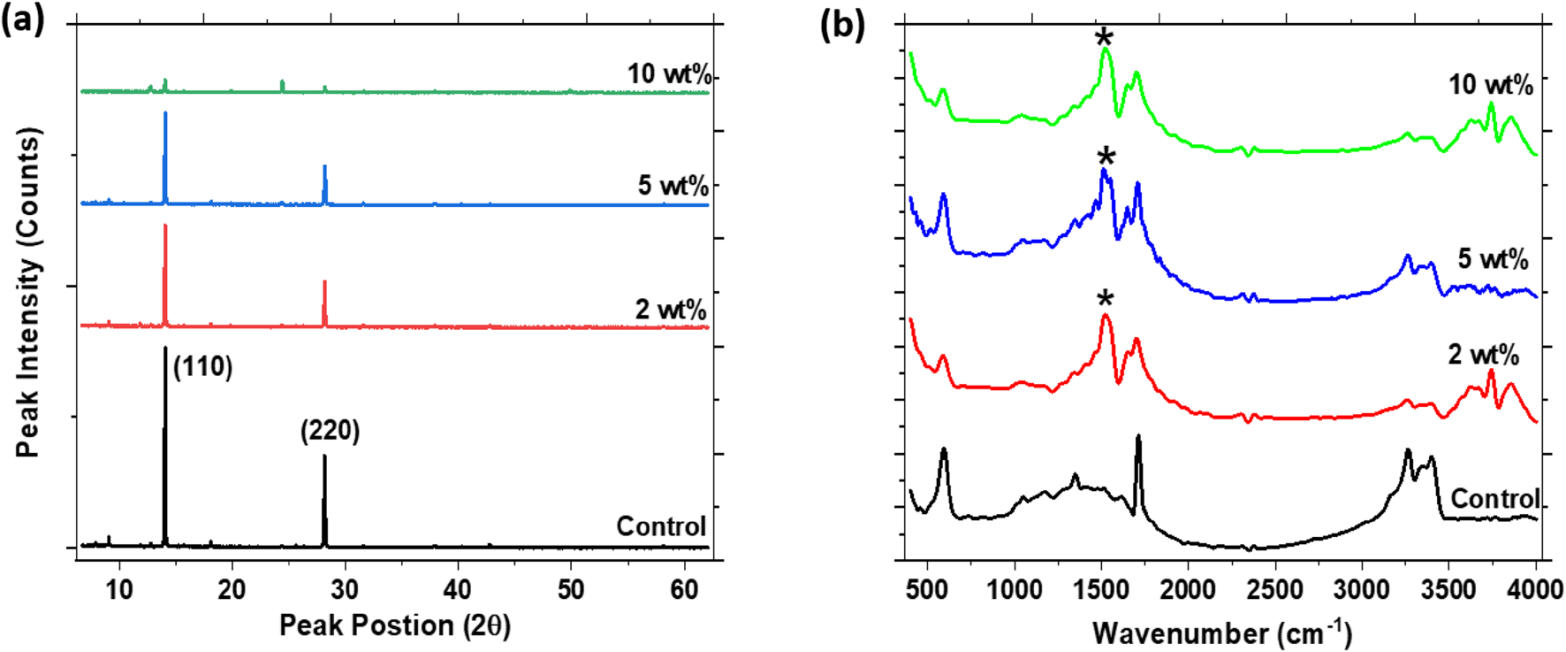
(**a**) XRD patterns, and (**b**) FTIR spectra of the perovskite films at different weight proportions of PEO.

Additives incorporated into perovskite films can influence the nucleation and growth kinetics of the crystals, thus affecting their microstructural properties. Some additives can cause metal-halogen-metal bond alteration and phase transformation in perovskite films through the filling of lattice vacancies and changes in the unit cell dimension^33^. In the case of the PEO additive, the XRD analysis of the perovskite films with and without PEO reveals that all the films crystallized in the cubic perovskite phase as indicated by their preferred orientation along the planes (110) and (220) in the XRD spectra of Fig. 3a. This means that PEO did not alter the phase of the perovskite film, despite the morphological differences observed in the SEM images in Fig. 1.

However, the PEO additive led to a reduction in the crystallinity of the perovskite film as confirmed by the gradual decrease in the intensity of the major peak at (110) as the weight proportion of PEO in the film increased. We also noted that PEO did not alter the crystal lattice parameter of the perovskite films since the position of the major peak did not show any observable shift. Therefore, the PEO additive may have interacted with the perovskite to form new chemical bonds with uncoordinated ionic species without necessarily changing its crystal structure.

To obtain more information on the interaction of PEO with the perovskite films, we carried out FTIR studies as a function of the wt% of PEO in the film. The FTIR spectra; as seen in Fig. 3b; shows the emergence of new peaks in the PEO-modified perovskite films at wavenumber (k) values in the range 1517–1528 cm^−1^, ascribable to the C–N stretch vibrational mode. The peak appears at k value of 1528 cm^−1^ for the film containing 2 wt% of PEO and red-shifts to 1517 cm^−1^ for the film with 10 wt% of PEO. Similarly, the peak signature for C=O stretch; appearing at 1711 cm^−1^; in the control film redshifts to 1700 cm^−1^ for perovskite with 10 wt% of PEO. This redshift in the position of the peaks is an indication of the coordination effects between functional groups in PEO with the perovskite crystals. The observation has been attributed, in prior work, to the weakening of the C=O double bond due to the coordination effect from the oxygen atom in the PEO^26,34^. Moreover, being a Lewis base, the same effect can arise from electron delocalization from the carbonyl (C=O) group due to the formation of Lewis base-acid adduct between the oxygen in the PEO and PbI_2_ in the perovskite^35^.

**Figure 4:**
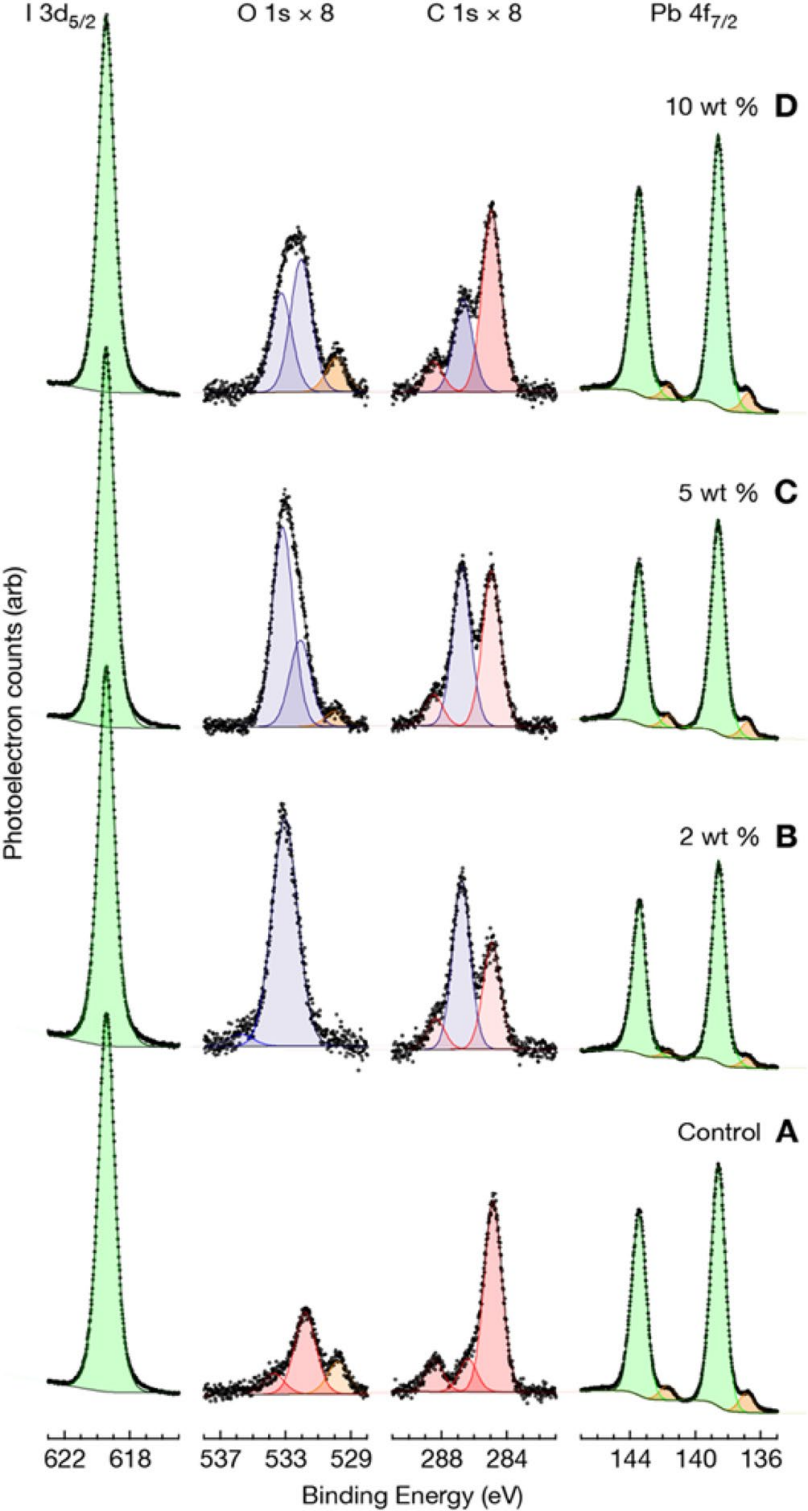
High-resolution XP spectra of perovskite films at different wt% of PEO (**A**) 0, (**B**) 2, (**C**) 5 and (**D**) 10.

Due to the possible interaction of PEO with uncoordinated atomic species in perovskite film, the surface chemical state of the film may change when PEO is introduced into it. To find out whether the PEO loading influenced the surface chemistry of the perovskite films, we probed the variation in the surface characteristics of the films using XPS. The high-resolution XP spectra of the perovskite film; as a function of the percentage weight of PEO; are presented in Fig. 4. It is seen that the XP spectra of all the films are nearly identical except for the additional features at binding energies of ~ 286 eV and ~ 532 eV (shaded blue) that are visible in the XP spectra of perovskite films modified with PEO. These features are those of the O 1s and C 1s core-level peaks which can be ascribed to PEO additive. The presence of these features in the PEO-modified perovskite films strongly reaffirms the interaction of PEO with the perovskite as earlier revealed by FTIR results of Fig. 3b. The features shaded in red are those of the adventitious and formamidinium carbon originating from the perovskite itself while the ones shaded in orange are those of reduced lead which are indicative of beam damage in the films.

**Figure 5:**
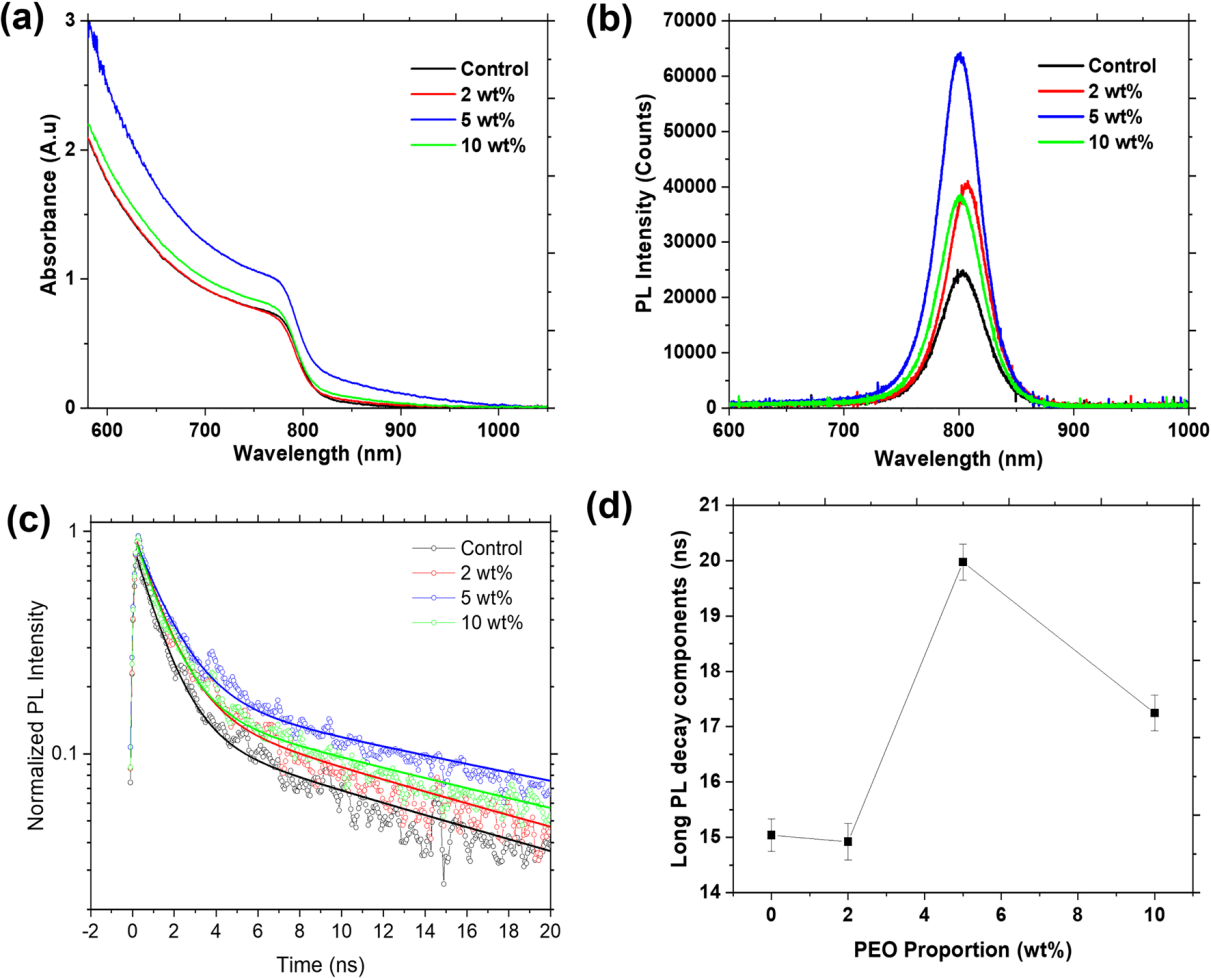
(**a**) UV–Vis spectra (**b**) steady stead PL, (**c**) TRPL and, (**d**) long decay lifetime as a function of wt% of PEO (Fluence 280 µJ/cm^2^).

The optical response of perovskite photoactive material is an important aspect in its PV application as it determines the photon absorption and photogeneration processes which are the backbone of solar to electricity conversion^36^. Most lead-based perovskite films have their light absorption edge around 800 nm which can either be extended or reduced with the incorporation of additives. We studied the optical response of the FA-rich perovskite film deposited on FTO substrate as a function of PEO wt% and the results are displayed in Fig. 5a–d. The optical absorption properties of the perovskite films (Fig. 5a) indicate that the absorbance of the films in the visible region improved with the wt% of PEO up to 5% and decreased thereafter. The increase in absorbance as the PEO content increase from 0 to 5 wt% is a consequence of the observed increase in the grain size of the perovskite films (Fig. S1). The decrease in absorbance when the PEO content is beyond 5 wt% is attributed to the microstructural changes that were observed in the perovskite films as per the SEM images in Fig. 1.

Photoluminescence (PL) properties and charge carrier lifetimes of the FA-rich perovskite films with and without PEO were studied using steady-state PL and time-resolved PL (TRPL) (Fig. 5b,c). Detailed interpretation of PL and TRPL of perovskite films and devices is non-trivial and requires consideration of multiple inter-dependent processes such as Auger recombination, bi-molecular recombination, trap-assisted Shockley–Read–Hall recombination, photon recycling, and carrier diffusion within the films and across interfaces^37–39^. Here, our goal is limited to elucidating the impact of PEO on carrier lifetime. We find that all the perovskite films exhibit a strong band edge emission peak at ~ 1.55 eV (800 nm) with a negligible shift as PEO content increases (Fig. 5b). We also observe that at the same excitation conditions (485 nm, 280 µJ/m^2^ 50 ps laser pulses with repletion rate of 10 kHz), PL intensity increases with increasing PEO content up to 5 wt% and decreases at 10 wt%.

Highest PL intensity (~ 3 times stronger compared to control) in the film with 5 wt% of PEO likely results from increased optical absorption of the excitation (Fig. 5a) as well as from a reduction in non-radiative defect-mediated recombination. This agrees well with its optimized morphological properties such as larger, more uniform grains, seen in the SEM micrographs in Fig. 1c. The TRPL measurements show PL kinetics within 20 ns time after optical excitation (Fig. 5c). Interpretation of PL kinetics in perovskite materials is complicated as mono-, bi- and trimolecular charge carrier recombination pathways are typically present simultaneously and depend on instantaneous carrier density^38^. At excitation fluence of 280 µJ/cm^2^, initial photoexcited carrier density is high, in excess of 10^19^ cm^−3^, and a combination of Auger and bimolecular recombination dominates at early times^38,40^. As carrier density drops below ~ 10% of its initial value, which we observe at times > 5 ns, bimolecular radiative recombination becomes dominant but carrier trapping contributes as well^38^. Trap-mediated monomolecular recombination becoming most probable only at carrier densities < 10^16^ cm^−3^, or < 0.1% of the initial carrier density in our experiments, which occurs outside our time-window. Experimental data in Fig. 5c are fitted to a double exponential decay. Fast decay component is 1.1 ± 0.1 ns, and is independent of composition. The long decay, which dominates the signal in 5–20 ns time window, changes with PEO content as shown in Fig. 5d. We can draw qualitative conclusions by analyzing changes in this long decay time. We find that the kinetics becomes slower with an increase in PEO content up until the 5 wt% (Fig. 5d). Presuming that radiative decay rate is the same in all films in this time window, we can then ascribe the change to a reduced contribution of carried trapping. This is probably driven by the increase in grain size and the corresponding reduction of the role played by the grain boundary trap states.

**Figure 6:**
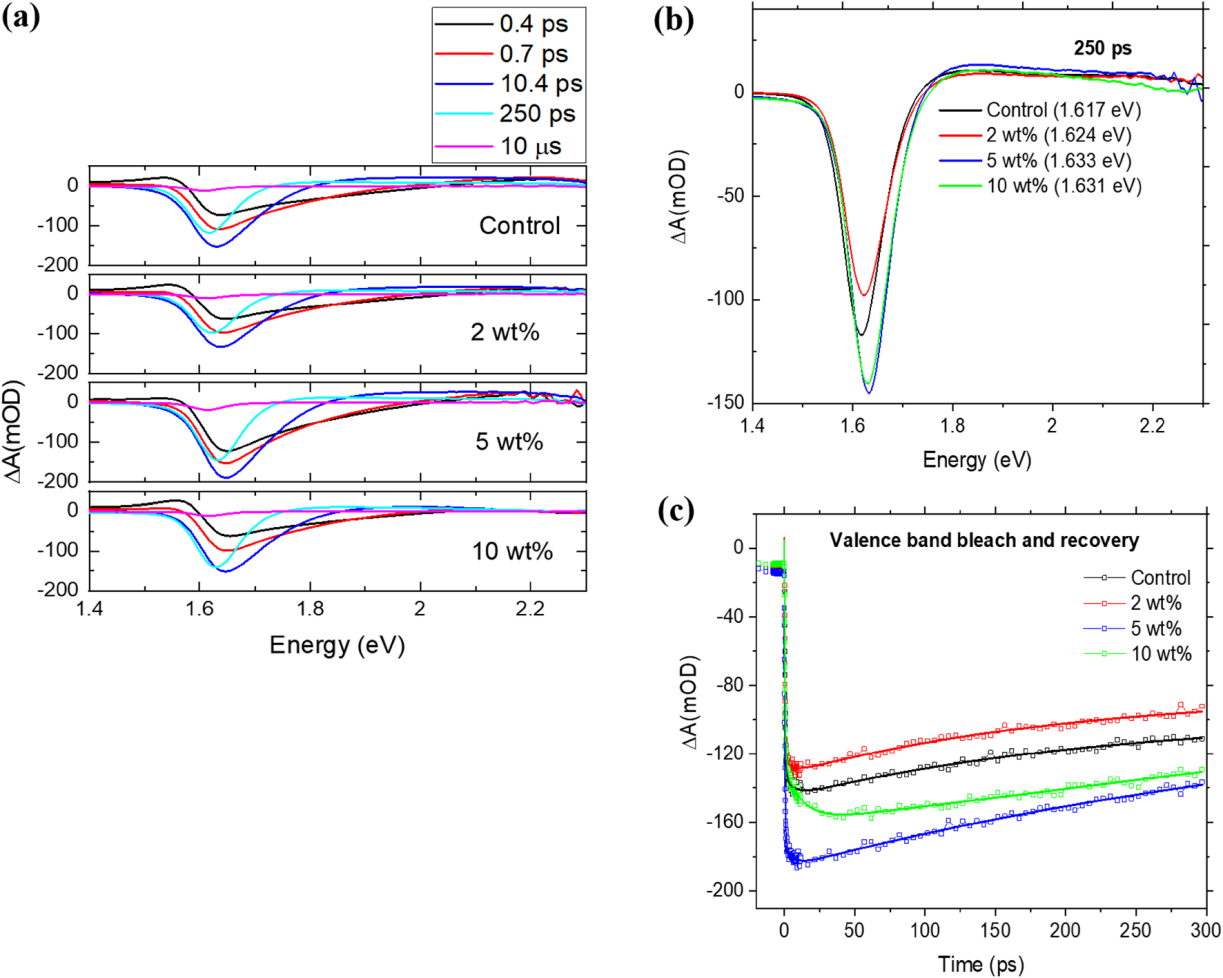
Transient absorption dynamics of the perovskite films with varying weight proportions of PEO: (**a**) TA spectra of the different perovskite films in the delay time range of 0.4–250 ps, (**b**) transient absorption spectra at a fixed pump-probe delay time of 250 ps and (**c**) bleach recovery kinetics of different perovskite films.

To obtain more insights into the optical properties of perovskite films, we studied the excited state dynamics of the FA-rich perovskite films over sub-picosecond to hundreds of picoseconds time scales by carrying out ultrafast TAS (Fig. 6). Figure 6a shows TA spectra of the different perovskite films in the delay time range of 0.4–300 ps following excitation with 50 fs, 485 nm pulse with a 51 µJ/cm^2^ fluence. Transient absorption spectra exhibit a negative band in the 1.61–1.63 eV range attributed to the bleaching of the absorption band edge due to the valence band depopulation^41–44^. It is also seen that the absorption band edge bleach peak shows a small redshift with increasing pump delay times (Fig. 6a). As Fig. 6b shows, transient absorption spectra at a fixed pump-probe delay time (here shown at 250 ps) are qualitatively similar in all the films. A small shift is seen as a function of PEO content, from (1.617 ± 0.002) eV to (1.633 ± 0.002) eV when the proportion of PEO is increased from 0 to 5 wt% and to (1.631 ± 0.002) eV for a weight proportion of 10%. This shift may be caused by the morphological changes in the film or intra-band excitonic states within the bandgap of the film^45^. Positive transient absorption bands on both sides of the bleach arise due to the excitation-induced changes in the refractive index.

Bleach recovery kinetics provides additional information on the optical behavior of the perovskite films (Fig. 6c). In films with PEO content up to 5 wt%, recovery is biexponential over the 300 ps experimental time window, with a slower component that cannot be resolved by TA but likely lasts for microseconds. In fact, the bleach is not fully recovered on the time scale given by the excitation and probe repetition rate of 100 kHz (10 µs), as shown in transient absorption spectra in Fig. 6a. The fast recovery component is similar in all three films at 5–7 ps. The slower component is ~ 200 ps for the control (0 wt%) and 2 wt% films, but it slows down to ~ 450 ps in the optimal performance film with 5 wt% PEO and is even slower (> 500 ps) in phase-segregated film (10 wt%). Through its interaction with the uncoordinated ionic species in the perovskite film, PEO can induce surface dipole formation or cause band bending between the bulk and surface of the perovskite films which will influence their electronic structure and the dynamics of charge carriers^46,47^.

**Figure 7:**
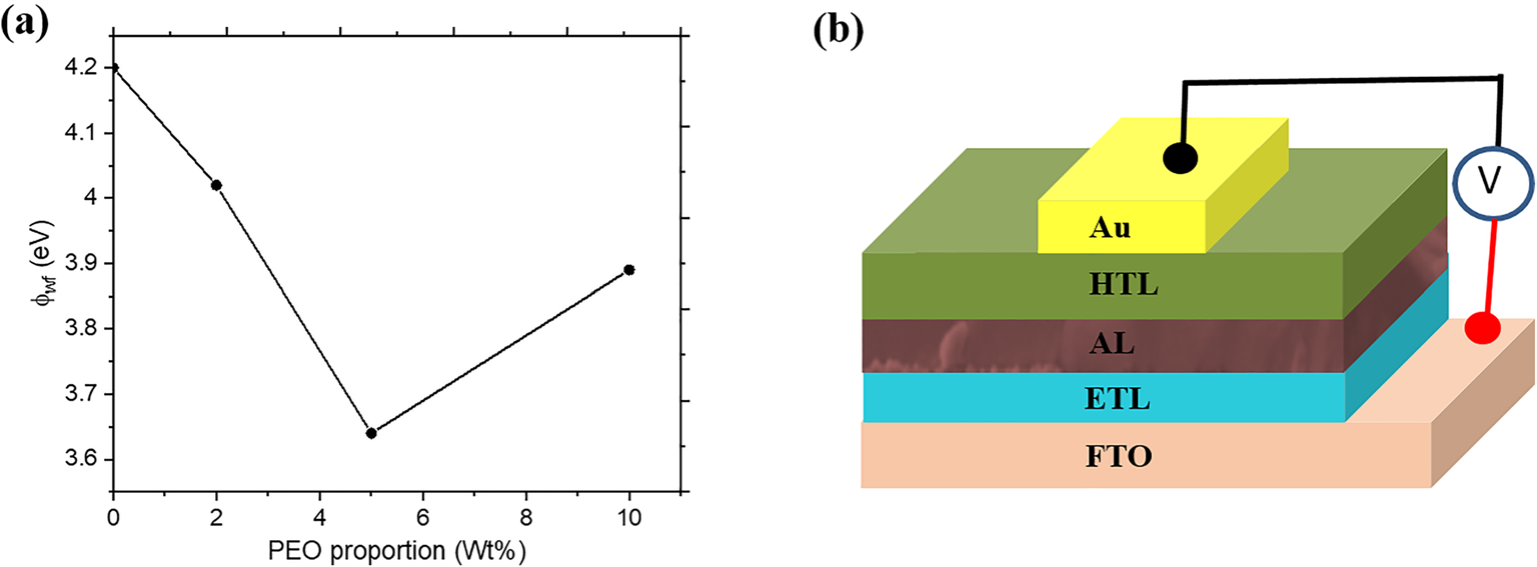
(**a**) Work function of perovskite films as a function of the weight proportions of PEO. (**b**) Architecture of the fabricated PSC device.

To develop a deeper understanding of the influence of PEO on the electronic band structure of the different FA-rich perovskite films, we carried out UPS studies on the films containing varied weight proportions of PEO. Figure 7a shows the variation of the surface work function (Φ_WF_) of the perovskite films with the wt% of PEO. We can see clearly that the value of Φ_WF_ decreased as the PEO proportion increases up to 5 wt% where it begins to show a slight increase. The decrease in Φ_WF_ is an indication of an upward shift in the position of the Fermi level (E_f_) which may possibly arise due to an increase in the donor density (n-type conductivity) resulting from the additional electrons from the electron-rich oxygen in PEO. The variation in Φ_WF_ with the weight proportion of PEO can have some influence on the interfacial charge transport between the AL and ETL/HTL in the fabricated planar PSC (Fig. 7b) which will affect its PCE.

## Performance characteristics of the PSC

**Figure 8:**
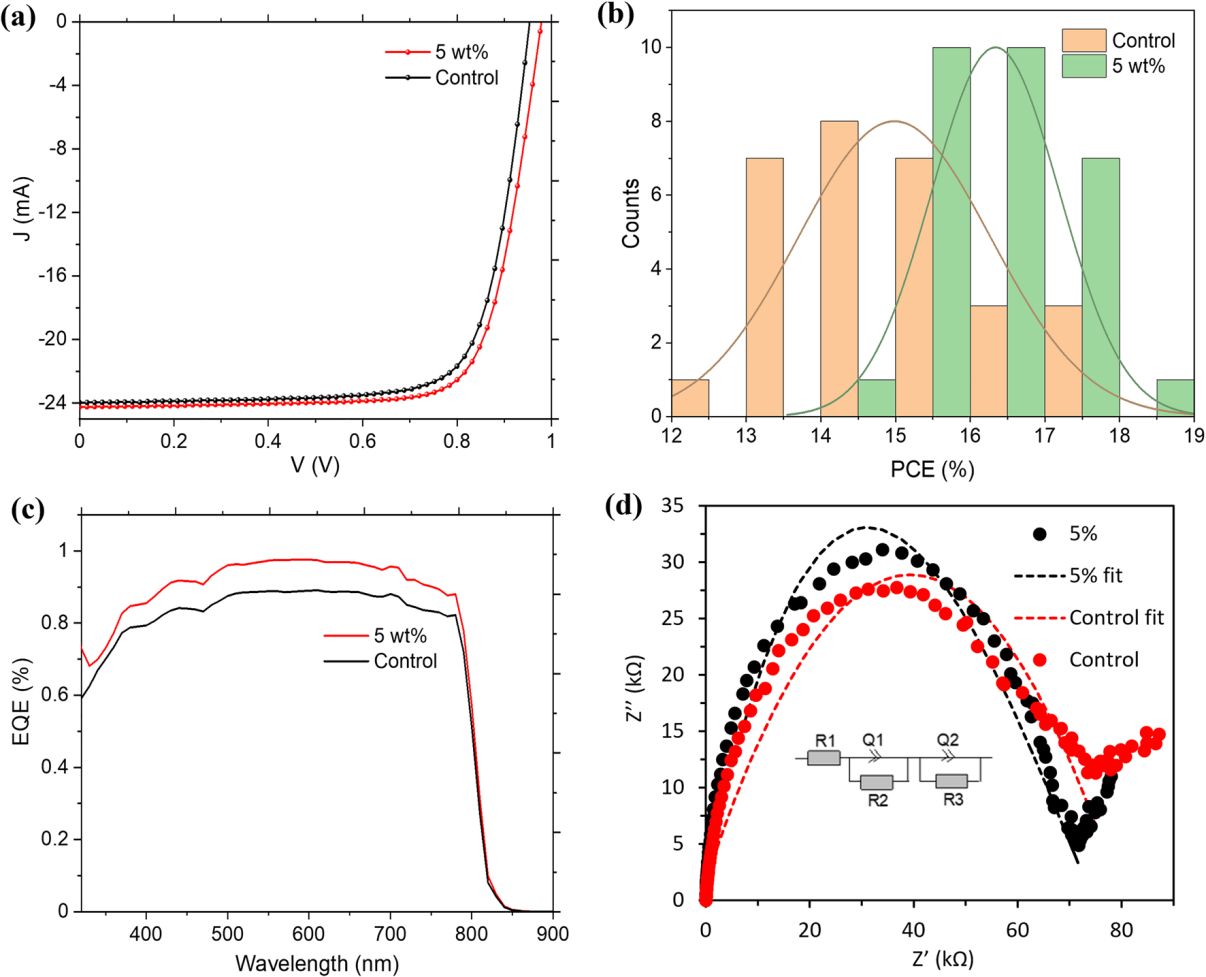
Comparison of (**a**) J–V curves, (**b**) statistical distribution of the PCE, (**c**) EQE, and (**d**) Nyquist plot for the control and PEO-modified PSC devices. The inset in (**d**) is the equivalent circuit of the fit.

Photovoltaic performance characteristics of PSCs are influenced by several factors which include the light absorption and charge transport properties of the photoactive layer. These factors influence the efficiency of charge carrier generation and collection which consequently affect the current density and photovoltage of the resulting PSC device. To investigate the effect of PEO on the photovoltaic performance characteristics of PSC, we fabricated a series of planar PSC devices with varying weight proportions of PEO in the active layer. We then studied the variation of the PV, electrochemical impedance, and charge carrier collection properties of the different PSCs with the proportion of PEO in the active layer. Figure 8a–d shows the J–V curves, EQE curves, and EIS of the champion devices for both the PEO-modified and the control PSCs. As depicted from Fig. 8a, the V_oc_ and J_sc_ values for the champion devices were slightly higher for the PEO-modified device when compared to those of the control device. The PCE for the champion device modified with 5 wt% of PEO in the AL was 18.03% while that for the control device was 17.34%. To assess the overall effect of the PEO additive on the PV performance parameters of the fabricated FA-Rich PSC, several devices with and without PEO were fabricated under the same conditions. Figure 8b shows the statistical analysis of the PCEs for a set of 29 devices with and without PEO. As can be seen from the figure, the peak of the normal distribution curve for the PEO-modified PSC devices is at a higher PCE value than that of the control device, an indication that the PEO-modified device exhibited better average PCE than the control device. The mean value of the PCE was 9.07% higher for the device in which 5 wt% of PEO was incorporated into its AL. A clear picture of the variation in the mean values of the PV parameters with the weight proportion of PEO is shown in Table S1 in the supplementary material. The variation in these parameters with the proportion of PEO in the AL can partly be attributed to the defect passivation and electronic band structure modification effects of PEO as revealed by the SEM and UPS studies.

PSCs are also known to exhibit J–V hysteresis which is caused by a number of factors including their defect states^48,49^. We studied the hysteretic behavior of the control and the PEO-modified PSC by recording their J–V curves under forward (F) and reverse (R) scan directions and comparing their hysteresis indices (HIs). The scans were carried out at the speed of 150 mV/s. Figure S2 shows the J–V curves under forward and reverse scans for both the PEO-modified and the control PSCs devices. The HIs of the PSCs were calculated from their PCEs in the reverse and forward scan directions^50^. The average HI for the PEO-modified PSC devices was 0.147 ± 0.019 while that of the control devices was 0.442 ± 0.146. Therefore, the PEO-modified devices exhibited smaller average values of HIs, an observation that has been ascribed to reduction in defect density as a result of the passivation effect of PEO^31^.

The modification of the perovskite AL in PSC is known to have an impact on its charge carrier dynamics^51^. To examine the effect of PEO additive on the charge carrier dynamics in the fabricated devices we compared the charge carrier collection efficiencies of the PEO-modified devices to that of the control device. The charge carrier collection efficiencies of the PSC devices at each wavelength are shown by the EQE curves of Fig. 8c. The EQE values of PSC with 5 wt% of PEO are generally higher than that of the control device at all the wavelengths under consideration. This means that the PEO-modified device has better charge carrier collection efficiency than the control device.

To find out the reason behind the observed differences in the charge carrier collection efficiencies of the PSC devices, we analyzed their electrochemical impedance characteristics in the dark. The results; expressed in the form of a Nyquist plot (Fig. 8d); indicate that the device with the PEO-modified AL had slightly higher recombination resistance and lower series resistance than the control device. The Nyquist curves for both the control and the PEO-modified PSCs were fitted using the Z fit tool in the EC lab software of BioLogic SP 300 potentiostat. The equivalent circuit of the fit is shown Fig. 8d inset. The values of the series resistance (R_1_) for the control and PEO-modified devices were respectively found to be 2.45 ± 0.34 Ω cm^2^ and 2.24 ± 0.22 Ω cm^2^ while the recombination resistance (R_2_) values were 25.07 ± 6.77 kΩ and 27.1 ± 4.31 kΩ. Therefore, the PEO-modified device exhibited slightly smaller values of R_1_ and slightly higher values of R_2_ when compared to the control device.

**Figure 9:**
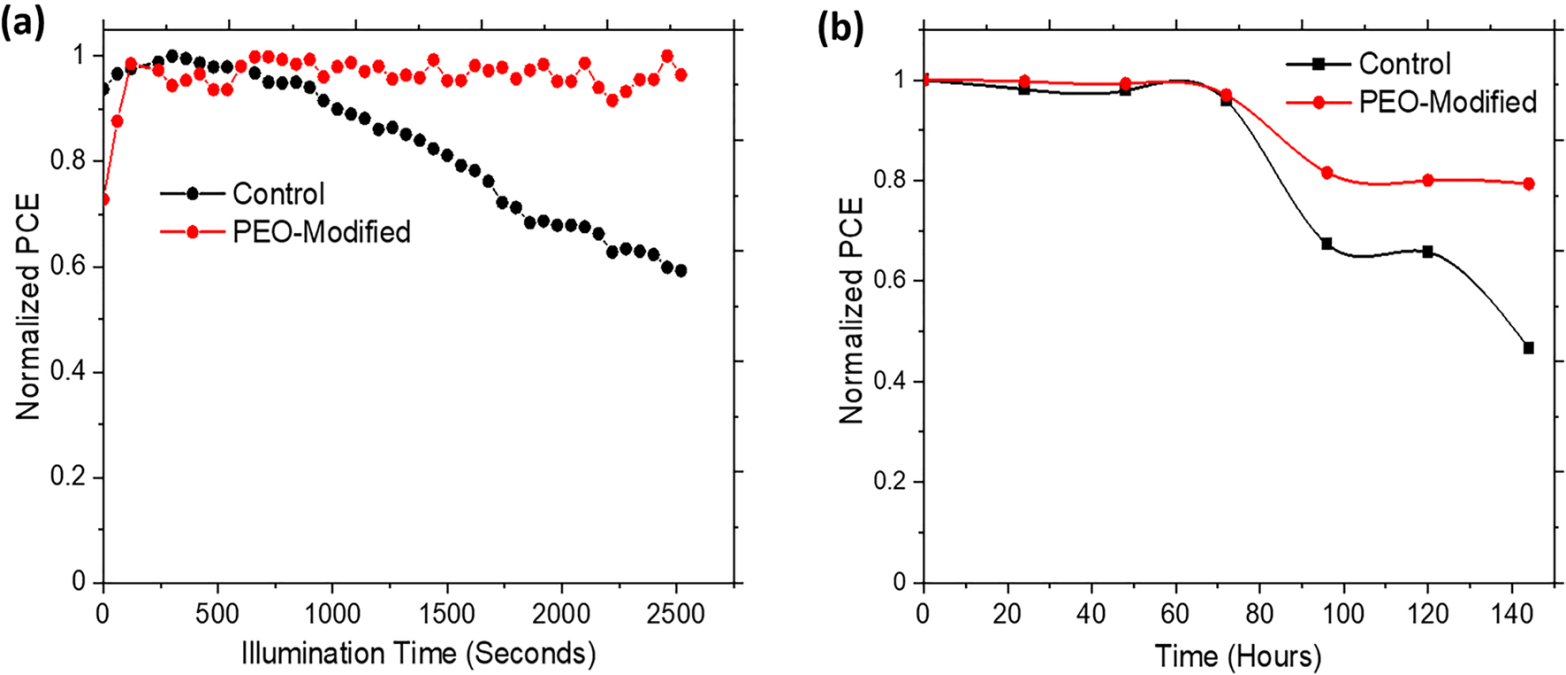
Stability of PSCs under (**a**) continuous illumination, (**b**) storage in ambient conditions.

The operational stability of PSC is another parameter for assessing its performance and is influenced by various intrinsic and extrinsic factors^52^. To understand the effect of PEO additive on the operational stability of PSCs, we analyzed the variation of the PCE of the control and PEO-containing devices under continuous illumination and when stored under ambient conditions without any form of encapsulation. Figure 9a,b shows the temporal variation in the normalized PCE of the devices under continuous illumination and storage in humid conditions. It is obvious from Fig. 9a that the PEO-modified device had its normalized PCE remaining almost constant during the 2500 s of continuous illumination while the normalized PCE for the control device showed a continuous decrease after about 500 s. Furthermore, it is seen in Fig. 9b that the PEO-modified device was able to retain about 80% of its initial PCE for up to 140 h of storage in an unencapsulated state while that of the control device dropped below 80% after just about 85 h. These results indicate that the PEO additive improves the light and moisture-induced degradation resistance of PSC. The possible reason behind the improved stability for the PEO-modified PSC is that the PEO particles slowed down the morphological changes in the perovskite film and led to reduction in charge trapping centers due to reduced grain boundary area resulting from the defect healing ability of PEO^29,53,54^. It is important to note that the enhanced stability of the PEO-modified PSCs can promote the scale-up of PSCs via spraying with device area of over 1 cm^2^. This shall be carried out in our future work on large area fabrication of PSCs for modules.

## Conclusion

The incorporation of the right proportion of PEO into the perovskite layer improves the optoelectronic properties of the film and modulates the interfacial energetics in PSCs. Consequently, the light absorption and charge carrier dynamics in PSC are enhanced leading to an improvement in the photocurrent and the PCE. A planar PSC whose AL was modified with 5wt% of PEO demonstrated better PCE and operational stability relative to the control device fabricated under the same conditions. The PCE of the champion device with 5 wt% in its AL was 18.03% while that of the control device was 17.34%. The higher PCE in the PEO-modified device originated from its reduced recombination that led to better charge carrier collection efficiency as revealed by the EIS and EQE studies. The PEO-modified device was able to retain over 80% of its initial PCE after being stored for about 140 h under humid conditions (average relative humidity of 62.5 ± 3.25%) without encapsulation.

## Materials and methods

### Materials

Unless otherwise stated, most of the materials used in this work were purchased from Sigma Aldrich and used as received. Apart from Polyethylene Oxide (PEO) polymer, all the other materials have been listed in our previous work on FA-Rich PSC^13,55–57^.

### Materials processing

After sequential cleaning of pre-patterned FTO-coated glass substrate in a detergent, acetone, and IPA, a compact electron transport layer (ETL) was formed from mixed titanium dioxide (TiO_2_) and tin (iv) oxide (SnO_2_) precursor solutions following the procedure described elsewhere^55^. The SnO_2_ and TiO_2_ in the ETL precursor solution was mixed in the volume proportion of 0.2 (optimized from previous work) before being spin coated onto the FTO coated glass substrate^57^.The perovskite active layer was formed on the ETL via a two-step spin-coating process where the lead (II) Iodide (0.5993 g in 1 ml of mixed DMF-DMSO solvent) was first spin-coated, followed by the organic components that were mixed with PEO in different weight proportions. The organic component consisted of a mixture of FAI, MACl and MABr in the ratio of 10:1:1 by mass (60 mg, 6 mg and 6 mg) respectively^13,55^. The weight proportion of PEO in the organic mixed precursor solution was varied from 0 wt% (control), 2 wt%, 5 wt%, and 10 wt% to study the effects of PEO weight percentage on the properties of perovskite/PEO composite films. After forming the perovskite films, a Spiro-OMeTAD-based hole transport layer (HTL) was deposited on it via spin-coating. The HTL was formed by spin coating a mixed solution consisting of 72 mg of Spiro-OMeTAD in 1 ml of chlorobenzene, 35 μL solution of LI-TFSI salt (260 mg in 1 ml of acetonitrile) and 30 μL of *t*BP over the perovskite layer. The LI-TFSI salt and *t*BP additive are important in enhancing the conductivity of Spiro-OMeTAD thus improving its hole extraction ability^58^. A final layer of gold (electrode) was then thermally evaporated onto the HTL to obtain a complete planar PSC.

### Materials characterization

The morphological and structural features of the prepared perovskite films were characterized, respectively, using a field emission scanning electron microscope (JEOL JSM-700F, Hollingsworth & Vose, MA, USA) and an X-ray diffractometer (Malvern PANalytical, Westborough, MA, USA). The optical properties and the charge carrier dynamics of the films were probed using techniques such as ultraviolet–visible spectroscopy, photoluminescence, ultrafast Transient Absorption Spectroscopy (TAS), X-ray Photoelectron Spectroscopy (XPS), and Ultra-Violet Photoelectron Spectroscopy (UPS). The TAS measurements of perovskite films were also carried out using a HARPIA-TA Ultrafast Transient Absorption Spectrometer powered by 1030 nm Ytterbium laser (Carbide, Light Conversion). Optical parametric amplifier (Orpheus, Light Conversion) generated 400 nm pump pulses with a fluence of 51 µJ/cm^2^ at the sample. The difference in absorption as a function of pump-probe delay time was detected by an Andor spectrograph and Si photodiode array. The SEM, XRD, UV–Vis, XPS and UPS characterizations were done on five different sets of perovskite films deposited on FTO-coated glass substrates and similar trends were observed in all the sets. For PL, TRPL and TAS, one set of samples were studied. The error in determining position of the bleach peak was based on peak analysis for each particular spectrum.

For the complete PSC devices, various characterization techniques were used to understand the effects of the PEO additive on its overall performance metrics. The current density–voltage (*J*–*V*), electrochemical impedance spectroscopy (EIS), and external quantum efficiency (EQE) characteristics of the fabricated PSC device were studied on a device area of 0.05 cm^2^ using a Keithley source meter, potentiostat (SP-300, BioLogic Instrument), and QuantX-300 quantum efficiency measurement system.

## Supplementary Information

Supplementary Information.

## Data Availability

All data generated or analyzed during this study are included in this published article and its supplementary information files.
