# Supplementary material for: Effects of polyethylene oxide particles on the photo-physical properties and stability of FA-rich perovskite solar cells
Source: Sci Rep. 2022 Jul 27;12:12860. doi: 10.1038/s41598-022-15923-y (PMC9329478; doi:10.1038/s41598-022-15923-y)
Supplement: Supplementary file 1 — Supplementary Information. [file 41598_2022_15923_MOESM1_ESM.docx]

**Effects of Polyethylene Oxide Particles on the Photo-Physical Properties and Stability of FA-Rich Perovskite Solar Cells**

Richard K. Koech^1,2,3^, Yusuf A. Olanrewaju^1,2^, Reisya Ichwani^2,4^, M. Kigozi^1^, Deborah O. Oyewole^2,4^, Omolara V. Oyelade^2,5^, Dahiru M Sanni^5^, Sharafadeen A. Adeniji^5^, Erika Colin-Ulloa^6^, Lyubov V. Titova^6^, Julia L. Martin^7^, Ronald L. Grimm^7^, Abdulhakeem Bello^1,5^, Oluwaseun K. Oyewole^2,4,*^, Esidor Ntsoenzok^8^ and Winston O. Soboyejo^2,4,9,*^

^1^Department of Materials Science and Engineering, African University of Science and Technology, Km. 10 Airport Road, Abuja, Nigeria.

*^2^Department of Mechanical Engineering, Worcester Polytechnic Institute, 100 Institute Road, Worcester, MA 01609, USA.*

*^3^Department of Mathematics, Physics and Computing, Moi University, P.O Box 3900-30100, Eldoret, Kenya.*

*^4^Program in Materials Science and Engineering, Department of Mechanical Engineering, Worcester Polytechnic Institute, 100 Institute Road, Worcester, MA 01609, USA.*

*^5^Department of Theoretical and Applied Physics, African University of Science and Technology, Km 10 Airport Road, Abuja, Nigeria*

*^6^Department of Physics, Worcester Polytechnic Institute, 100 Institute Road, Worcester, MA 01609, USA.*

*^7^Department of Chemistry and Biochemistry, Life Science and Engineering Center, Worcester Polytechnic Institute, 100 Institute Road, Worcester, MA 01609, USA.*

^8^ *CEMHTI-CNRS Site Cyclotron, 3A rue de la férollerie, 45071 Orléans, France.*

*^9^Department of Biomedical Engineering, Worcester Polytechnic Institute, 60 Prescott Street, Gateway Park Life Sciences and Bioengineering Center, Worcester, MA 01609, USA.*

*^*^Corresponding author*

**Fig S1**


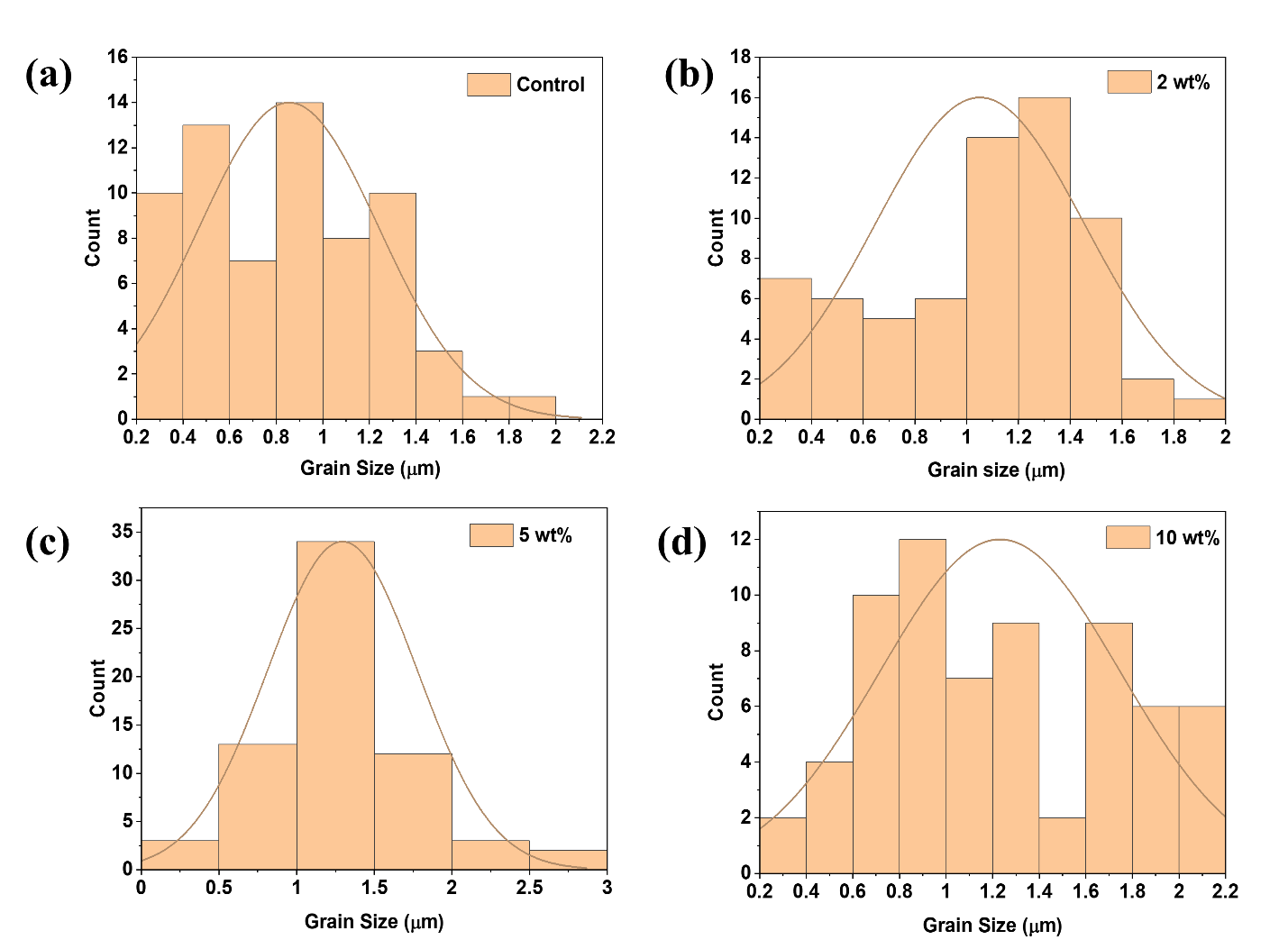


Figure S1. Grain size distribution for perovskite films with (a) 0 (b) 2 (c) 5 and (d) 10 wt% of PEO

Table S1. PV performance parameters for PSCs with different PEO content in the AL

| **Wt% of PEO** | **PCE** | **J_sc_** | **V_oc_** | **FF** |
| --- | --- | --- | --- | --- |
| 0 | 17.34 (14.98 ± 1.29) | 24.54 (23.15 ± 0.77) | 0.992 (0.929 ± 0.04) | 76.60 (69.76 ± 5.75) |
| 2 | 16.57 (15.56 ± 0.84) | 23.71 (23.0 ± 0.84) | 0.992 (0.966 ± 0.03) | 73.10 (70.05 ± 3.12) |
| 5 | 18.03 (16.34 ± 0.86) | 24.52 (23.64 ± 0.56) | 1.01 (0.970 ± 0.02) | 76.15 (71.25 ± 2.87) |
| 10 | 14.5 (13.12 ± 2.07) | 22.61 (21.45 ± 1.48) | 0.992 (0.963 ± 0.05) | 66.68 (63.12 ± 4.65) |
| *Minimum of 8-10 devices were tested to estimate parameters.* | | | | |


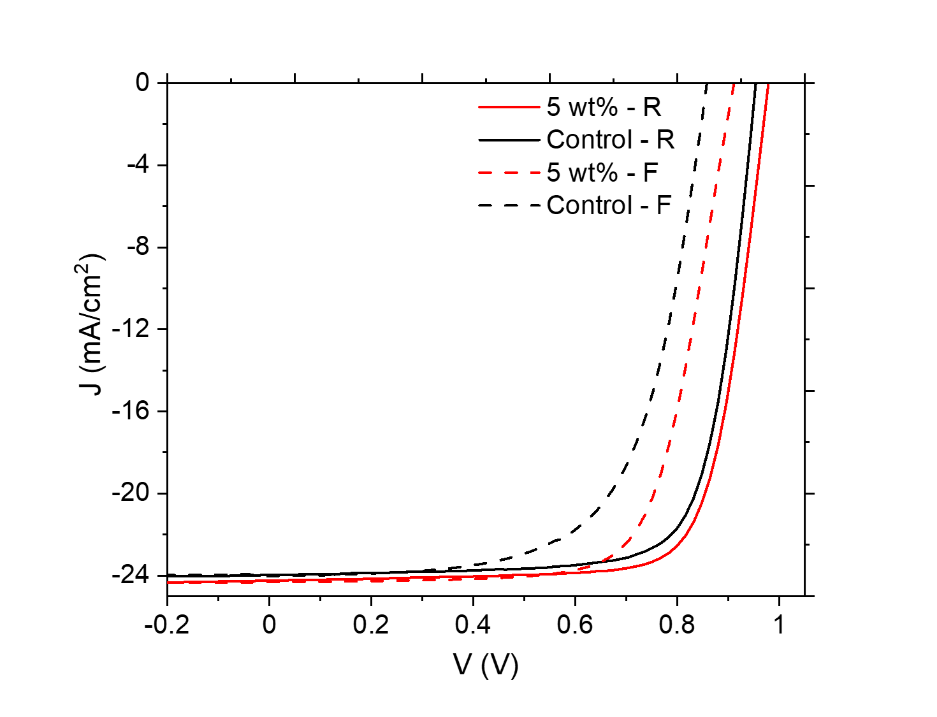


Figure S2. J-V curves of the control and the PEO-modified PSC devices under reverse and forward bias scans
